# Perspective: Are Online Food Delivery Services Emerging as Another Obstacle to Achieving the 2030 United Nations Sustainable Development Goals?

**DOI:** 10.3389/fnut.2022.858475

**Published:** 2022-03-03

**Authors:** Si Si Jia, Alice A. Gibson, Ding Ding, Margaret Allman-Farinelli, Philayrath Phongsavan, Julie Redfern, Stephanie R. Partridge

**Affiliations:** ^1^Engagement and Co-design Hub, School of Health Sciences, Faculty of Medicine and Health, The University of Sydney, Sydney, NSW, Australia; ^2^Menzies Centre for Health Policy and Economics, Sydney School of Public Health, Faculty of Medicine and Health, The University of Sydney, Sydney, NSW, Australia; ^3^Charles Perkins Centre, The University of Sydney, Sydney, NSW, Australia; ^4^Prevention Research Collaboration, Sydney School of Public Health, Faculty of Medicine and Health, The University of Sydney, Sydney, NSW, Australia; ^5^Nutrition and Dietetics Group, Faculty of Medicine and Health, The University of Sydney, Sydney, NSW, Australia; ^6^The George Institute for Global Health, The University of New South Wales, Sydney, NSW, Australia

**Keywords:** online food delivery, sustainable development goals, global health, public health, systems approach

## Abstract

Online food delivery usage has soared during the 2019 novel coronavirus (COVID-19) pandemic which has seen increased demand for home-delivery during government mandated stay-at-home periods. Resulting implications from COVID-19 may threaten decades of development gains. It is becoming increasingly more important for the global community to progress toward sustainable development and improve the wellbeing of people, economies, societies, and the planet. In this perspective article, we discuss how the rising use of these platform-to-consumer delivery operations may impede advances toward the United Nations 2030 Sustainable Development Goals (SDGs). Specifically, online food delivery services may disrupt SDGs that address good health and wellbeing, responsible consumption and production, climate action and decent work and economic growth. To mitigate potential negative impacts of these meal delivery apps, we have proposed a research and policy agenda that is aligned with entry points within a systems approach identified by the World Health Organization. Food industry reforms, synergised public health messaging and continuous monitoring of the growing impact of online food delivery should be considered for further investigation by researchers, food industry, governments, and policy makers.

## Introduction

Unhealthy diets, non-communicable diseases, urbanization, and climate change are recognized as significant challenges to global health (1). The United Nations have urged countries to act on 17 Sustainable Development Goals (SDGs) across economic, social and environmental dimensions to promote healthy lives and wellbeing and make cities inclusive, safe, and sustainable by 2030 (2). Online food delivery services (OFDS) are potentially impeding our progress toward the SDGs—impacting the way we eat, work, and care for the environment. Defined as “platform-to-consumer delivery operations” of ready-to-consume meals, OFDS offer delivery of a wide variety of takeout foods and beverages from kitchens to doorsteps (3).

The OFD industry is now widespread across the globe and big multinational corporations are dominating the market. Billion-dollar companies such as UberEats, DoorDash, and Just Eat operate in thousands of cities and show no sign of slowing down. Globally, OFDS market revenue increased by 27% in 2020, reaching $136.4 billion USD (4). These services are likely to proliferate further, as UberEats estimates that despite the return to dine-in restaurants, consumers are now spending three times more on OFDS compared to pre-pandemic levels (5). Furthermore, Just Eat or otherwise known as Menulog, reported a 79% increase in total orders between 2020 and 2021, across its 17 operating countries including UK, Germany, Canada, and Netherlands (6).

Considering the growing prevalence and market influence of OFDS, it is important to track the impact of OFDS on key public health challenges, such as increasing accessibility of unhealthy foods, promotion of excessive consumption, poor working conditions of delivery couriers in a gig economy and the environmental implications of takeout food packaging. This perspective piece will discuss how OFDS may disrupt progress toward SDGs that address good health and wellbeing, responsible consumption and production, climate action and decent work and economic growth.

## Junk Food on Demand: Impact on Nutrition, Health, and Wellbeing

OFDS may pose a considerable risk to the aim of SDG 3 to “Ensure healthy lives and promote wellbeing for all at all ages”. Research has shown that these OFDS have an abundant offering of menu items that are of poor nutritional quality. From an investigation of 680 popular food outlets on the market leading OFDS in Sydney, Australia, 37.6% (256/680) of popular outlets were classified as a “fast-food franchise” store, and out of the 2,463 most popular menu items identified, 2,358 (95.7%) were identified as “discretionary foods” (7), characterized as high in saturated fat, sodium and sugar, and not essential for health.

Moreover, the two leading OFDS (UberEats, Menulog) in Australia are partnered with the top 10 fast food franchise stores (Subway, McDonalds, Dominos, KFC, Hungry Jacks, Red Rooster, Nando's, Pizza Hut, Zambero, Oporto) (8). This signifies the dominance of fast-food franchise outlets on these platforms which are now another additional avenue for consumers to access their menu items. It is well-known that offerings from fast food franchise outlets are “energy-dense and nutrient poor” (9) and evidence has highlighted the strong association between high fast-food consumption and obesity (10). Furthermore, research has shown that diets high in inflammatory foods such as refined grains, sugary drinks, processed meats and other “junk foods”, have been associated with increased inflammation in the body and can elevate subsequent risk of heart disease by 46% and stroke by 28% (11). These highly inflammatory foods are widely available on these online food delivery platforms as shown in findings from a recent cross-sectional study (12). From over 196 independent takeaway food outlets available on UberEats in Sydney, Australia, discretionary cereal-based mixed meals was the largest category found within complete menus (42.3%, 5849/13,841). These include foods such as pizzas, burgers, pides, pasta, wraps, and sandwiches (12).

A Canadian study analyzed the full menus of retailers partnering with a large OFDS and similarly found low Healthy Eating Index-2015 scores—ranging from 19.95 to 50.78 out of 100 (with a score of 100 being the healthiest) (13). This study also found a mean delivery distance of 3.7 km or 2.3 miles measuring from postal codes in Ontario to online food retailers (13). Australian research has likewise, demonstrated that the mean delivery distance from food outlet to suburbs was 3 km, and around 90% of delivery distances were greater than 1 km (7)—a distance that typically defines the neighborhood food environment. The neighborhood food environment reflects the spatial extent of an individual's typical shopping behavior which could be reasonably walked by an adult in 15–20 min (14). As such, these platform-to-consumer services may be expanding local neighborhood food environments.

Altogether, findings suggest OFDS are expanding the traditional definition of the neighborhood food environment, increasing the accessibility of food outlets which mostly offer items with poor nutritional quality.

## Over-Consumption and Excess Promotion

In addition to increasing accessibility of food outlets, OFDS further encourage excessive consumption with aggressive marketing and promotion tactics. Macromarketing researchers are wary of the current marketing systems which promote an era of excess as business models choose to “create” rather than “address” consumer needs, without consideration of the waste generated from overall consumption (15). OFDS may add further burden to unsustainable practices of mass consumerism which threaten progress toward SDG 12: “Ensure sustainable consumption and production patterns”.

UberEats and Menulog frequently distribute promotional vouchers that offer free meals, discounts, and free delivery (16, 17). These are often disseminated through emails to past customers signed up to the OFD platform (18) or handed-out in person at high-traffic locations such as train stations (19). Moreover, there is evidence that OFD companies “COVID-wash” their social media promotions—a practice where companies align themselves with social or health issues of COVID-19 to enhance their own image (20). In a content analysis of Instagram posts from leading OFDS in 2020 during the pandemic, the most used COVID-19 marketing strategy was related to “combatting the pandemic” (76/123, 62%) (21). This theme helped brands position themselves to be “in this together” and encourage consumers to “support their local businesses”. These findings were echoed in another content analysis study conducted in New Zealand. The most used theme in 36% of all COVID-19 related social media posts intended to generate feelings of community support during the challenging time. Fast-food brands were also found to be the largest proponents of COVID-washing, accounting for 46% of all COVID-19 posts (20).

Furthermore, during the pandemic, an increase in social media posts promoting “junk foods” from leading OFD brands was observed. In a recent study, we found junk foods accounted for 69.1% of all food and beverage items featured, compared to 58.3% in 2019 (21). Similarly, a study from Brazil indicated widespread presence of unhealthy food advertising as ultra-processed beverages such as soft drinks were among the most shown in advertisements for OFDS throughout COVID-19. Free delivery also prevailed in advertisements of junk food items such as ice cream, candy, high sodium snacks, and pizza (22). More research found that menus offering unhealthy meals had more photos and discounts compared to meals offering unprocessed and/or minimally processed foods (23). Taken together, OFDS continue to facilitate fast-food delivery at heavily discounted prices and excess promotions, perpetuating the culture of excessive consumption.

## Unsustainable Plastic Waste and CO2 Emissions

Plastic waste is a key global environmental concern with annual plastic consumption currently at over 300 million tons, which is expected to double in the next 20 years (24). High volumes of online food delivery consumption exacerbates plastic waste and adds to the increasing contamination of natural environments such as the ocean, freshwater systems, and terrestrial areas (25). Subsequently, OFDS may have a huge climate cost and are another impediment to SDG 13: “Take urgent action to combat climate change and its impacts”.

Takeout meals ordered from OFDS can come with extensive quantities of plastic material, namely food containers, cutlery, napkins, and plastic bags among others (26). These materials are often single-use, requiring large quantities of energy and raw materials to produce, transport, and be disposed (27). A study on the environmental impacts of takeout food containers revealed that single-use polypropylene containers are the worst packaging material for takeout food, with many negative impacts on the environment (26). In China, researchers found that the total amount of packaging waste from food delivery surged from 0.2 million metric tons in 2015 to 1.5 million metric tons in 2017 (28). Plastic containers made from polypropylene and polystyrene foam accounted for approximately 75% of the total food delivery packaging waste in weight. COVID-19 lockdowns further aggravated China's plastic waste dilemma: during the lockdown in Wuhan, an average of 130,000 takeout orders were made per day, which totaled to more than 279,500 m of lunchboxes over a 6-week period (29).

Excessive energy consumption and carbon emissions are associated with the waste produced from food delivery. Based on annual online food delivery data of 179.2 million active users, a 2016 Chinese study found an average ordering frequency of 2 times/week and average delivery distances of 25 km (30), which resulted in an estimated Green House Gas (GHG) emission of 73.89 Gigatonnes (Gt) carbon dioxide-equivalent emissions (CO_2eq_) (30). In Australia, COVID-19 lockdowns led to a 20% increase in household solid wastes, partly due to a surge in food deliveries which contributed sizeable amounts of paper, plastic packaging waste and single-use waste (31). Another study found in 2018, the disposal of single use packaging from online food orders in Australia led to 5,600 tons of CO_2eq_ (32). With online food orders expected to increase to 65 million in 2024, researchers project a 132% rise in carbon emissions to 13,200 tons of CO_2eq_ (32). As such, the environmental threat of OFDS is progressively evident and needs to be considered as governments globally move toward carbon emission reduction targets.

## Fuelling the Gig Economy

Instead of steering toward the SDG 8: “Promote sustained, inclusive and sustainable economic growth, full and productive employment and decent work for all”, OFDS stimulate the gig economy and may veer away from sustainable economic growth that will create quality jobs. While OFDS have facilitated new job opportunities and increased flexibility of work, the quality of these jobs is questionable with little-to-no employment rights and poor work health and safety conditions (33).

Advances in online technology have fuelled the rise of the “gig economy”—a free market system in which mobile apps or websites connect consumers with individual workers providing services. Gigs are denoted by short-term, one-off employment contracts mediated by online platforms which include online food delivery. Although spending on gig economy in Australia declined severely during the period of early COVID-19 lockdown restrictions in March 2020, it increased to 40% above pre-lockdown levels. This growth was almost entirely driven by the online food delivery sector, which itself increased by more than 100% between August and October 2020 (34). Indeed, UberEats Australia, a leading OFDS, has reported providing 59,000 work opportunities during 2020 which is an eight-fold increase since 2016 (5).

In a report on digital platform work in Australia, it has been revealed that food delivery workers choose to work with OFDS for flexibility and to supplement existing income streams (35). However, food delivery workers are more dependent on income generated from meal delivery compared to gig workers on other digital platforms (35). This report also suggests food delivery workers were more likely to work longer hours in a week and were more likely to say the income was essential for meeting basic needs (35). Moreover, food delivery platforms may vary in their contractual agreements where workers may be independent contractors rather than employees. This places workers at risk of insecure income, no insurance, personal or paid leave, no workers compensation, superannuation or certain taxes. Over a long term, gig work as a food delivery worker may be financially untenable. A gig worker who spends 5–10 years in the gig economy full time, could potentially be $40,000–$100,000 AUD worse off in accumulated superannuation at retirement compared to a minimum wage earner (34). Major reforms of OFD work in the gig economy are required to increase the quality of these jobs, improve the livelihood of workers and be sustainable in the long term.

## Discussion

Existing reports shows achieving the 2030 UN SDGs will require tremendous efforts ahead by governments and industries globally given the considerable setback induced by the COVID-19 pandemic (36). A systems approach to the complexities of public health issues has been proposed by notable researchers—as outlined in the 2011 and 2015 Lancet Series on Obesity (37, 38) and has been a developing research area to inform the National Institute for Health and Care Excellence guidelines on obesity prevention (39). The synergising of goals and targets within and between systems affecting health including manufacturing, financial, transportation, and food, may be essential to meaningful progress. The EAT-Lancet commission on Food, Planet, and Health is an example which shows the power of goal alignment (40). This report has outlined the role of diet with human health and environmental sustainability—addressing both the rise in unhealthy diets, the targets of the UN Sustainable Development Goals and the Paris Agreement. As research on the impacts of OFDS is still in its infancy, robust solutions to resolving the issues outlined in this perspective have yet to be developed. However, using a similar systems-based approach, the following calls to action identify areas for existing systems to merge.

## Proposed Calls to Action

The World Health Organization (WHO) (41) has now acknowledged the growing impact of online food environments on people's diet choices. In a recently published report, WHO Europe has proposed the use of a systems approach to make informed decisions on potential interventions and/or regulation of these OFDS or otherwise known as “Meal Delivery Apps” (MDA). Taking a systems approach, several entry points to change were identified. These include Nutrition, Physical Activity, Alcohol consumption, Labor, Road Safety, Food Safety which are key elements that use existing mechanisms to solve complex issues (41). The entry points align with the SDGs discussed in this perspective and may benefit from collective action by food industry, governments, policy makers and researchers.

**Figure 1:**
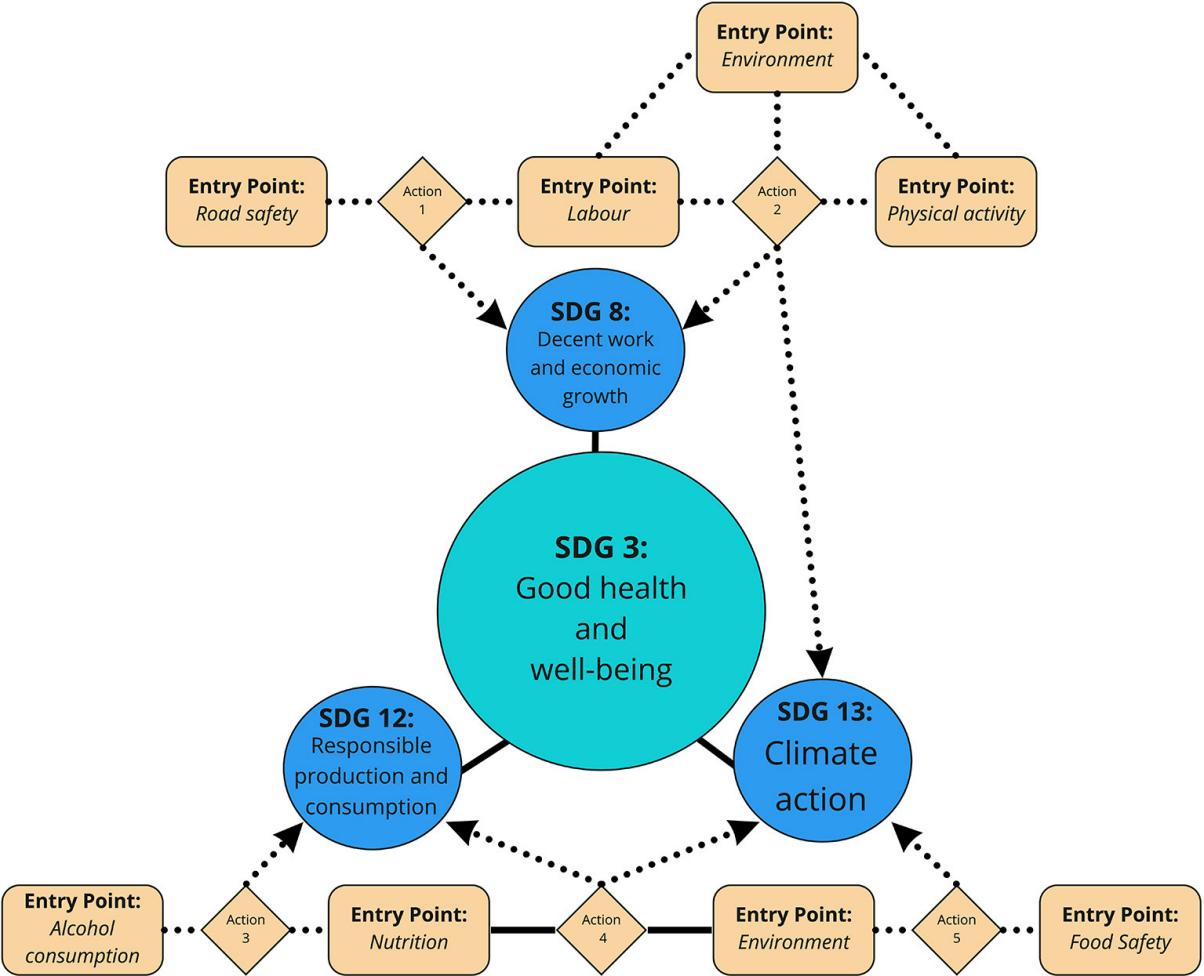
Conceptual diagram identifying areas for entry points and Sustainable Development Goals relating to online food delivery services to merge, forming action points to ultimately address SDG 3 Good Health and Wellbeing. Actions are defined and described in detail in Table 1.

In Table 1, we propose a research and policy agenda with action points that address SDGs and entry points from the WHO report. We have also illustrated examples of current and emerging research across these action points. Figure 1 was designed to demonstrate how these action points can work together to strive toward SDG 3: “Good Health and Wellbeing” for the benefit of public health. The proposed action points may also later converge with recommendations outlined from WHO Europe's commentary piece (58).

**Table 1:** Proposed action points to mitigate negative impacts of online food delivery services and address the 2030 UN Sustainable Development Goals and entry points identified in the WHO Meal Delivery Apps Report.

**Action**		**UN sustainable development goal**	**WHO MDA entry point**	**Current and emerging research**
1	Advocate for major reforms to the gig economy sector to recognize full employment rights for food delivery workers to improve working conditions	SDG 3 SDG 8	Labor, Road Safety	Menulog Australia has started a pilot employment program around Sydney's Central Business District—giving their worker's rights to a minimum wage and superannuation contributions by directly employing them (42)/ New Zealand's second-largest private sector trade union, “FIRST Union” has launched a class action lawsuit against Uber to seek better conditions and security of rideshare and food-delivery work (43).
2	To create healthier neighborhood food environments by developing cycle-friendly or convenient and walkable pathways within transport networks and city infrastructures.	SDG 3 SDG 8 SDG 13	Labor, Physical activity, Environment	To improve potential access to healthier foods and reduce the reliance on getting takeaway meals delivered, a “15-min” city may be a possible solution. Proposed by Moreno and colleagues, a “15-min city” is an urban planning concept that advocates for the provision of basic urban amenities at distances that would take local citizens no more than 15 min to access by foot or by bicycle (44). This urban planning concept has inspired many cities to embrace becoming more cyclable and walkable. The city of Paris is leading the “15-min city” transformation (45) while other cities have also seen notable action such as Melbourne (46), Shanghai (47), Singapore (48), Portland (49), and Bogota (50).
3	Disincentivise unhealthy food and beverage choices through regulatory pressure to limit promotions such as monthly subscriptions and junk food advertising on social media while promoting healthier food options on these platforms.	SDG 12 SDG 3	Alcohol consumption, Nutrition	Research on the monitoring of social media food marketing content targeted toward children and adolescents is still in its infancy. However, this is a high priority for global bodies such as the World Health Organization. Further research may benefit by aligning with the proposed tools and initiatives from WHO to monitor food and beverage marketing to children via television and the Internet (51). In addition, the UK government has announced a ban on junk food advertising online and before 9 p.m. on TV from 2023 (52). This ban will affect all paid-for forms of digital marketing—from Facebook ads to paid search results on Google, text message promotions, and paid activity on sites such as Instagram and Twitter. More work should also focus on the potential benefits of online food delivery services—as many healthier food options can be promoted and selected instead. Digital nudging interventions have shown promising results with improvement to the nutritional quality of online school canteen lunch orders (53). Intervention participants had significantly lower energy content and saturated fat content than control lunch orders.
4	Generate clear public health messaging on both the nutritional quality and environmental impacts of OFDS usage in relation to sustainability.	SDG 3 SDG 12 SDG 13	Nutrition, Environment	Adding nutritional labeling requirements to online food delivery service platforms may be a feasible policy option to inform individuals of the nutritional quality of menu items. The UK has expanded its menu calorie-labeling policies to all restaurants, cafes and takeaways—including online food delivery and this will be implemented in April 2022 (54). Ecolabeling, a method of identifying products or services that are environmentally preferable (55), may also be a potential avenue to changing individual dietary choices while also advocating for environmental sustainability. A recent systematic review has shown promising results to suggest ecolabeling is associated with the selection and purchase of more sustainable food products (56).
5	Investigate sustainable and food safe takeaway packaging options and implement across food outlets partnered with OFDS	SDG 3 SDG 13	Environment, Food safety	Research on sustainable packaging is also growing and there have been suggestions that reusable packaging systems have better environmental benefits over single-use systems. There is an increasing number of companies now providing alternatives to single-use cups for restaurants and cafes—CupClub (U.K.), Meu Copo Eco (Brazil), Globelet (Australia), ReCup (Germany), and Revolv (Indonesia). Companies such as GoBox (US), recircle (Switzerland), Returnr (Austria), Ozarka, and Sharepack (The Netherlands) are leasing reusable containers to restaurants, cafes, bars, and food trucks. It remains unclear however, how this could be applied to all online food delivery outlets considering the need for customers to return empty packaging that will be cleaned and refilled for future use (57).

As research on the impact of OFDS continues to grow, ongoing monitoring and evaluation is critical to the development of policy options for regulating the digital food environment. Dashboards are considered as useful tools to help users visualize and understand complex information in a snapshot. They have been developed to monitor global food systems (59) and food environments (60) and may also be essential to tracking progress of online food delivery services. We therefore also propose the inclusion of online food settings within existing monitoring and evaluation frameworks of food systems and food environments (60).

## Conclusion

In a world now grappling with ongoing repercussions of the COVID-19 pandemic, resulting societal and environmental changes exacerbated by COVID may further derail the trajectory toward meeting the SDGs set by the United Nations. OFDS are likely to proliferate, providing valued convenience in an increasingly fast-paced modern society. However, the potential disruption to our health and the environment is substantial, interfering with overarching SDGs. Food industry reforms synergised public health messaging and continuous monitoring of the growing impact of OFDS may be part of the solution to collectively address the issues of sustainability, environmental health, decent work and economic growth and nutrition. Multidisciplinary action and research are urgently needed to further investigate such solutions.

## Data Availability Statement

The original contributions presented in the study are included in the article/supplementary material, further inquiries can be directed to the corresponding author/s.

## Author Contributions

SJ and SP contributed to the conceptualization of the manuscript. SJ wrote the first draft of the manuscript and designed all tables and figures included in submission. SP provided primary supervision of SJ, reviewing the first draft of the manuscript. AAG, DD, PP, MA-F, and JR edited and reviewed the manuscript drafts. All authors contributed to manuscript revision, read, and approved the submitted version.

## Funding

SJ was supported by the Australian Government's Research Training Program Stipend Scholarship, AAG receives funding from the National Health and Medical Research Council, NSW Health and Diabetes Australia, JR receives fellowship and research grants from the National Health and Medical Research Council, NSW Health, and Medical Research Future Fund, DD receives funding from the National Health and Medical Research Council and NSW Health, MA-F receives funding from the National Health and Medical Research Council, Australian Research Council, NSW Health and Cancer Council NSW, PP receives funding from NSW Health, SP receives funding from the National Health and Medical Research Council, National Heart Foundation, NSW Health and the Medical Research Future Fund.

## Conflict of Interest

The authors declare that the research was conducted in the absence of any commercial or financial relationships that could be construed as a potential conflict of interest.

## Publisher's Note

All claims expressed in this article are solely those of the authors and do not necessarily represent those of their affiliated organizations, or those of the publisher, the editors and the reviewers. Any product that may be evaluated in this article, or claim that may be made by its manufacturer, is not guaranteed or endorsed by the publisher.
